# Women’s Sexual Empowerment and Its Relationship to Contraceptive Use in Bangladesh: Findings From a Recent National Survey

**DOI:** 10.3389/ijph.2023.1606143

**Published:** 2023-10-19

**Authors:** Nishi Khatun, Sihab Howlader, Md. Mosfequr Rahman

**Affiliations:** Department of Population Science and Human Resource Development, University of Rajshahi, Rajshahi, Bangladesh

**Keywords:** women’s empowerment, sexual empowerment, contraceptive use, decision-making, empowerment indicators

## Abstract

**Objectives:** This study aimed to assess the relationship between women’s sexual empowerment and contraceptive use among married Bangladeshi women from a nationally representative sample.

**Methods:** Secondary data analysis was conducted using the Bangladesh Demographic and Health Survey (BDHS) 2017–18. The investigation covered a total of 14,515 married, non-pregnant women who were residing with their spouses. Multivariable logistic regression analysis was fitted to assess the relationship between the variables of interest.

**Results:** A unit increase in the sexual empowerment scale increases the odds of contraceptive use by 13%. While increasing age, being Muslim, having a spouse who is older by more than 10 years, and living in rural areas are associated with lower odds of using contraceptives than their respective counterparts, secondary or higher levels of education, having more living children, exposure to TV or radio, and employment are associated with higher odds of using contraceptives.

**Conclusion:** The study’s findings point to the need for addressing women’s perceptions of their right to sexual and reproductive health and equity in order to further efforts to achieve universal access to reproductive health services.

## Introduction

Women’s empowerment has become a subject of serious concern and has received tremendous attention to the researchers and policymakers in both developed and developing countries as a means of achieving development goals: poverty eradication, attaining universal primary education, achieving sustainable development, and enabling universal access to healthcare [1]. Since the International Conference on Population and Development (ICPD) in Cairo in 1994, women’s reproductive and sexual rights and empowerment have received special attention, and women’s empowerment has been recognized as important to their access to reproductive health services, including family planning. Women’s ability to decide on family planning methods and have an open discussion about them with their husbands or partners is restricted by their lack of power [2]. An empowered woman is more likely to use contraception to limit her family size because she has the power to manage resources and make decisions in her own best interests [3, 4]. It is also substantially linked to greater use of reproductive health services [5, 6], lowering fertility [7], reducing unintended pregnancy [8], and extending birth intervals [9].

Empowerment is complex and multidimensional in nature (political, economic, interpersonal, sexual, sociocultural, and so on), which is defined and conceptualize in various ways [3–5]. The World Bank defines empowerment as the “expansion of freedom of choice and action to shape one’s life” [10]. Kabeer defines it as a “process by which those who have been denied the ability to make strategic life choices acquired such an ability” [11]. However, women’s empowerment is frequently defined as a process and outcome, by which individuals gain power over their lives and decisions [12]. To be empowered, a woman must have the agency (i.e., perceived and actual self-efficacy and decision-making control) to move from making planned choices to achieving one’s self-determined goals, in addition to having key assets (e.g., health and education) and access to opportunities (e.g., employment) [13, 14]. At the individual level, empowerment entails women utilizing their assets, opportunities, and agency to make deliberate decisions and engaging in behaviors to alter life circumstances.

The relationship between women’s empowerment and contraceptive use is evident in a considerable amount of literature globally [2, 15–19]. However, most of these studies have estimated the effect of either a single-aspect dimension or an overall empowerment on contraceptive use, yet analysis has shown that not all dimensions of women empowerment are associated equally with contraceptive use [19]. Studies on the relationship between contraceptive use and women’s empowerment in relation to sexual context (sexual empowerment) are rare in the literature. A study from Ghana documented that an increasing level of sexual empowerment was significantly associated with an increasing use of contraceptives [20]. Furthermore, the widely recognized Sexual Relationship Power Scale (SRPS) acknowledges that power exists in relationships and within a larger social context; however, literature on the relationship between general power dynamics in an intimate relationship and contraceptive use suggests inconsistent findings, which may lead to the hypothesis that general relationship power may not accurately reflect a partner’s empowerment in a sexual context [21–24]. Since a woman may be empowered in one sphere of her life but not in other [22, 25], research into the relationship between sexual empowerment and contraceptive use is necessary, particularly in countries with pervasive gender power inequities.

The family planning programs in Bangladesh draw attention to the researchers and policymakers around the world because of the remarkable increase in contraceptive prevalence rate (CPR). In Bangladesh, there has been a significant increase in the CPR among women of reproductive age: from 8% in 1975 to 62% in 2017–18 [26]. According to the Bangladesh Demographic and Health Survey 2017–18, the CPR gradually increased from 1993 to 94 to 2007 but then stagnanted from 2011 to 2017–18 [26]. As a result, the declining trend in total fertility rate (TFR) has also been stalled at 2.3 children per woman from 2011 to 2017–18. The 4th Health, Population and Nutrition Sector Program (HPNSP) 2017–22 of the Government of Bangladesh has the aim of increasing the contraceptive prevalence rate (CPR) from 62% to 75% by the year 2022 in order to limit population growth and further improve maternal and child health [26]. To maintain the increasing trend of the CPR, the government of Bangladesh needs to identify the barriers to contraceptive usage and hence adapt an evidence-based pragmatic approach to its family planning programs. Therefore, researchers and policymakers have shown interest in exactly which factors affect contraceptive prevalence in Bangladesh. This study’s aim is to analyze data from a nationally representative sample of married women to assess the relationship between women’s sexual empowerment and contraceptive use.

## Methods

### Data

This study used data from the latest Bangladesh Demographic and Health Survey (BDHS), collected during the years 2017–19, conducted under the authority of the National Institute for Population Research and Training (NIPORT), Health Education and Family Welfare Division of the Ministry of Health and Family Welfare under the Training, Research and Development operational plan of the 4th HPNSP. Mitra and Associates implemented the survey with technical assistance from the ICF International. The survey followed a two-stage stratified sampling procedure. A total of 20,250 households were surveyed from 675 clusters (250 in urban areas and 425 in rural areas). In total, 20,250 households were surveyed following a two-stage stratified sampling procedure from 675 clusters in the first stage. A systematic sample of 30 households per enumeration unit was chosen in the second stage. However, because of extreme flood during the data collection, a survey of 20,160 households from 672 clusters was possible. There were 20,376 eligible women in these selected households. Of them, successful interview was completed from 20,127 ever-married women aged 15–49 years. Details of the survey methodology can be found elsewhere [26]. However, this study included women who were currently married, not pregnant, and living with their husbands at the time of the interview. Women with missing data on any of the key variables of interest were excluded, resulting in an analytic sample of 14,493. The BDHS 2017–18 was approved by the International Institutional Review Boards at ICF and the Bangladesh Medical Research Council (BMRC). The BDHS conformed to international ethical standards of confidentiality, anonymity, and informed consent. Due to the use of retrospective publicly available data, this study did not require further ethical approval.

### Measures

#### Current Contraceptive Use

Current use of contraception was defined as the proportion of women who reported that they were currently using a family planning method. Methods of contraception were categorized as modern method (pill, iud, injections, male condom, female sterilization, male sterilization, implants/Norplant, lactational amenorrhea, emergency contraception, other modern method), traditional method (withdrawal, other traditional), and folkloric method (other traditional). Women who did not identify as using any contraception method during the survey were categorized as currently non-users of contraceptives.

#### Sexual Empowerment

In this study, women’s sexual empowerment was investigated through the development of a crude composite score that reflected women’s perceptions of their ability to express themselves in sexual decision-making as well as their right to self-determination and equity in sexual relations. For the assessment of women’s sexual empowerment, four dichotomous items were used. These included (i) Can you say no to your husband if you do not want to have sexual intercourse? (ii) Could you ask your husband to use a condom if you wanted him to? (iii) If a wife knows her husband has a disease that she can get during sexual intercourse, is she justified in asking that they use a condom when they have sex? (iv) In your opinion, is a husband justified in hitting or beating his wife if she refuses to have sex with him? Adding the affirmative responses of the items (i), (ii), (iii), and the negative response of the item (iv) yields a score from 0 to 4. Reliability of this scale was evaluated by calculating the average correlation of the four binary items using the Kuder-Richardson Formula 20 method.

#### Covariates

A number of variables, including respondent’s age, education, religion, number of living children, age difference of the spouses, household wealth, working status, mass media access, living environment, and region, were included in this analysis as confounders. Age was categorized as 15–24, 25–34, and 35–49 years. Educational attainment of women as well as their husbands was categorized into two categories: no or primary education, and secondary or higher. The age gap between the spouses was classified as <5, 5–10, or ≥11 years. Household wealth was estimated using household assets and material possessions and classified into quintiles. Region was based on the location of the household within Bangladesh’s eight nationally recognized administrative divisions (Barisal, Chattogram, Dhaka, Khulna, Mymensingh, Rajshahi, Rangpur, and Sylhet). Living environment was categorized as urban or rural. Respondents’ mass media access was assessed by examining their exposure to three forms of mass media: reading newspapers/magazines, listening to the radio, and watching TV. For all cases, the response options were: not at all, less than once a week, and at least once a week. The other measures indicated respondents’ religiosity (Muslim or non-Muslim) and current working status (not working or working).

### Statistical Analysis

Descriptive statistics were calculated for the sample, and chi-square tests were used to identify bivariate associations between the variables of interest and contraceptive use. We also used either one-way analysis of variance (ANOVA) or Student’s *t*-tests to assess the bivariate relationship between different variables and the sexual empowerment score. As follow-ups to a significant ANOVA, Tukey honestly significant difference (HSD) *post hoc* multiple comparison tests were employed where appropriate. Association between women’s sexual empowerment and contraceptive use was estimated through a multivariable logistic regression analysis adjusted for the selected covariates. Variables that were found to be significantly associated either with contraceptive use or sexual empowerment score in bivariate analysis were included in the logistic regression model as covariates. The odds ratio (OR) and corresponding 95% confidence intervals (95% CIs) were estimated with statistical significance defined as α ≤ 0.05. The multicollinearity of the variables was checked by examining the variance inflation factors; in all cases, the values were less than 2.0, suggesting low multicollinearity. svy set of commands was used in Stata 14 (StataCorp, LP, College Station, TX, United States) to account for sample weighting related to the complex design of the DHS.

## Results

The median age of the women (*n* = 14,493) was 32 years [interquartile range (IQR) = 25–39 years]. As shown in Table 1, half of the women (50.2%) had a secondary or higher level of education, 89.5% identified themselves as Muslim, 54.9% had exposure to TV for at least once a week, and 69.8% were living in an environment classified as rural. More than one-third of the women (39.4%) reported that they had three or more living children, while 28.9% reported that their husbands’ ages differed by more than 10 years (≥11) years.

**TABLE 1:** Demographic profile of the study participants, Bangladesh demographic and health survey, Bangladesh 2017-2018 (*n* = 14,493).

Characteristics	Number	Percent^a^	95% CI
Age (years)			
15–24	3426	23.9	23.1–24.8
25–34	5145	35.6	34.7–36.5
35–49	5922	40.5	39.5–41.1
Educational attainment			
No or primary	7120	49.8	48.4–51.2
Secondary or higher	7373	50.2	48.8–51.6
Husband’s educational attainment			
No or primary	8000	56.5	55.1–57.9
Secondary or higher	6493	43.5	42.1–44.9
Religion			
Non-muslim	1592	10.5	8.8–12.6
Muslim	12901	89.5	87.4–91.2
No. of living children			
0	1019	7.0	6.6–7.6
1	3033	20.5	19.7–21.3
2	4777	33.1	32.0–34.1
≥3	5664	39.4	38.2–40.6
Age difference of the spouses, years			
<5	3336	23.1	22.2–24.0
5–10	6946	48.0	47.1–48.9
≥11	4211	28.9	28.0–29.9
Household wealth			
Bottom quintile	2814	19.2	17.6–20.8
Second quintile	2834	20.2	19.1–21.3
Third quintile	2740	19.7	18.6–20.8
Fourth quintile	2886	20.4	19.1–21.3
Top quintile	3219	20.6	19.1–22.2
Exposure to newspapers/magazine			
Not at all	12958	90.8	90.0–91.4
Less than once a week	921	5.8	5.4–6.3
At least once a week	614	3.4	3.0–3.8
Exposure to radio			
Not at all	13816	95.5	95.0–95.9
Less than once a week	392	2.7	2.3–3.0
At least once a week	285	1.9	1.6–2.2
Exposure to TV			
Not at all	5300	36.0	34.1–37.9
Less than once a week	1257	9.1	8.5–9.8
At least once a week	7936	54.9	53.1–56.8
Current working status			
Not working	7120	48.7	46.7–50.7
Working	7373	51.3	49.3–53.3
Living environment			
Urban	5554	30.2	29.2–31.2
Rural	8939	69.8	68.8–70.8
Region			
Barisal	1467	5.3	5.0–5.6
Chattogram	1804	15.4	14.6–16.1
Dhaka	2171	25.6	24.6–26.7
Khulna	2004	12.3	11.8–12.9
Mymensingh	1654	8.2	7.6–8.9
Rajshahi	1970	14.9	14.2–15.6
Rangpur	1896	12.7	12.0–13.3
Sylhet	1527	5.6	5.3–6.0

BDHS, bangladesh demographic health survey; CI, confidence interval.

^a^ In estimating percentages, the complex survey design and sampling weights were taken into account. Percentages may not total 100.0 because of rounding.

As displayed in Table 2, we observed that respondents age, educational level, number of living children, age difference of the spouses, media access, working status, living environment, and region were significantly associated with current contraceptive use as well as sexual empowerment score. The prevalence of contraceptive use was higher among women aged 25–34 years (81.9%), non-Muslims (79.2%), had ≥3 living children (76.0%), and age difference of the spouses 5–10 years (76.6%) than their respective counterparts. Women who reported exposure to TV at least once per week and were working were more likely to use contraception than those who had no exposure to TV (75.3% vs. 72.9%) and were not working (76.2% vs. 72.8%), respectively. Table 2 also presents that the mean sexual empowerment score was higher among women aged 15–24 years [mean (SD): 3.71 (0.62)], who had secondary or higher educational attainment [3.76 (0.55)], had one living child [3.73 (0.59)], had exposure to newspapers [3.86 (0.42)], radio [3.81 (0.50)], and TV [3.70 (0.62)] for at least once a week, and lived in an urban environment [3.70 (0.61)] than their respective counterparts. The Tukey HSD *post hoc* multiple comparison tests were employed for significant ANOVA, and the results are presented in Supplementary Table S1.

**TABLE 2:** Percentages of current contraceptive use and sexual empowerment score by different sociodemographic variables, Bangladesh demographic and health survey, Bangladesh 2017–2018 (*n* = 14,493).

Characteristics	Contraceptive use		Sexual empowerment score	
	n (%^a^)	*p*-value (χ^2^-test)	Mean (SD)	*p*-value (F-test/*t*-test)
Age (years)		<0.001		<0.001
15–24	2,533 (73.7)		3.71 (0.62)	
25–34	4,223 (81.9)		3.69 (0.61)	
35–49	4,048 (68.5)		3.54 (0.75)	
Educational attainment		<0.001		<0.001
No or primary	5,143 (72.5)		3.50 (0.76)	
Secondary or higher	5,661 (76.5)		3.76 (0.55)	
Husband’s educational attainment		0.804		<0.001
No or primary	5,960 (74.5)		3.54 (0.74)	
Secondary or higher	4,844 (74.6)		3.74 (0.57)	
Religion		<0.001		0.280
Non-Muslim	1,249 (79.2)		3.65 (0.64)	
Muslim	9,555 (74.0)		3.63 (0.68)	
No. of living children		<0.001		<0.001
0	396 (38.5)		3.66 (0.67)	
1	2,209 (72.8)		3.73 (0.59)	
2	3,900 (81.5)		3.68 (0.62)	
≥3	4,299 (76.0)		3.53 (0.74)	
Age difference of the spouses, years		<0.001		<0.001
<5	2,566 (76.5)		3.66 (0.65)	
5–10	5,321 (76.6)		3.66 (0.65)	
≥11	2,917 (69.5)		3.56 (0.73)	
Household wealth		0.109		<0.001
Bottom quintile	2,136 (76.3)		3.51 (0.77)	
Second quintile	2,103 (74.1)		3.58 (0.71)	
Third quintile	2,004 (73.0)		3.61 (0.68)	
Fourth quintile	2,182 (75.3)		3.66 (0.65)	
Top quintile	2,379 (74.1)		3.77 (0.54)	
Exposure to newspapers/magazine		0.438		<0.001
Not at all	9,628 (74.4)		3.61 (0.69)	
Less than once a week	706 (76.3)		3.79 (0.52)	
At least once a week	470 (75.6)		3.86 (0.42)	
Exposure to radio		0.325		<0.001
Not at all	10,283 (74.4)		3.62 (0.68)	
Less than once a week	84 (78.2)		3.79 (0.55)	
At least once a week	72 (74.7)		3.81 (0.50)	
Exposure to TV		0.013		<0.001
Not at all	3,862 (72.9)		3.55 (0.74)	
Less than once a week	957 (76.0)		3.57 (0.71)	
At least once a week	5,985 (75.3)		3.70 (0.62)	
Current working status		<0.001		0.002
Not working	5,196 (72.8)		3.64 (0.67)	
Working	5,608 (76.2)		3.61 (0.68)	
Living environment		0.040		<0.001
Urban	4,218 (75.8)		3.70 (0.61)	
Rural	6,586 (74.0)		3.59 (0.71)	
Region		0.006		<0.001
Barisal	1,105 (74.9)		3.65 (0.68)	
Chattogram	1,355 (75.0)		3.58 (0.72)	
Dhaka	1,636 (75.1)		3.69 (0.62)	
Khulna	1,476 (73.2)		3.65 (0.67)	
Mymensingh	1,233 (73.6)		3.68 (0.64)	
Rajshahi	1,464 (74.1)		3.61 (0.68)	
Rangpur	1,467 (77.5)		3.61 (0.67)	
Sylhet	1,068 (69.1)		3.56 (0.71)	

BDHS, bangladesh demographic health survey.

^a^ In estimating percentages, the complex survey design and sampling weights were taken into account.

**FIGURE 1:**
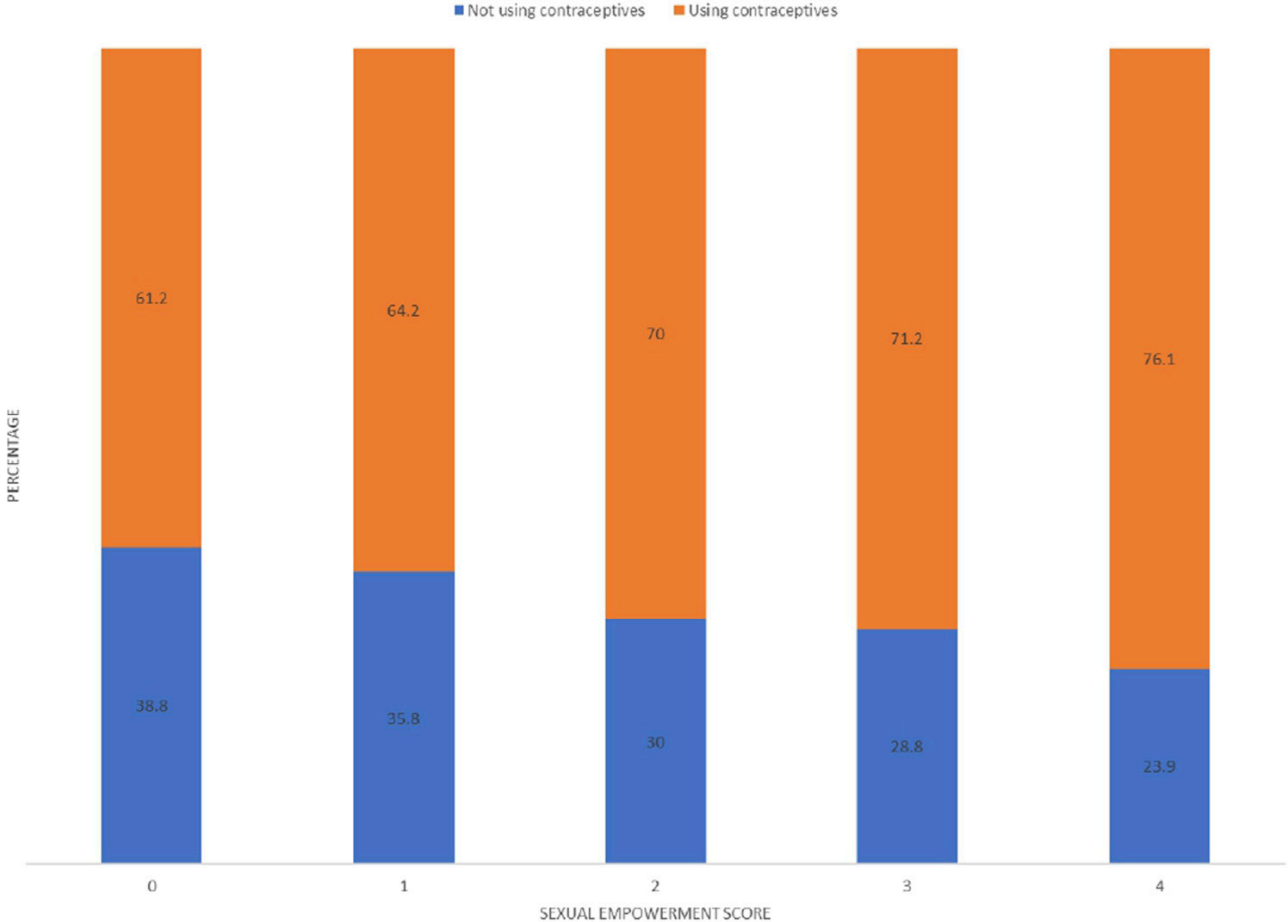
Prevalence of contraceptive use by women’s empowerment score, Bangladesh demographic and health survey 2017–18.

While we assessed the relationship between individual sexual empowerment variables and contraceptive use, two out of four sexual empowerment variables were found to be significantly associated (Table 3). The odds of contraceptive use were significantly higher among women who can refuse sex [unadjusted odds ratio (UOR): 1.17; 95% confidence interval (CI): 1.04–1.31], and women who can ask husbands to use condoms (UOR: 1.50; 95% CI: 1.35–1.67) than women who cannot refuse sex and cannot ask husbands to use condoms, respectively. Figure 1 illustrates the prevalence of contraceptive use by sexual empowerment score and demonstrates how the use of contraception increases as the sexual empowerment score increases.

**TABLE 3:** Percentage and odds ratio of current contraceptive use by sexual empowerment variables, Bangladesh demographic and health survey, Bangladesh 2017–2018.

Sexual empowerment variable	n (%^a^)	Contraceptive use		
		n (%^a^)	*p*-value (χ^2^-test)	UOR (95% CI)
Respondent can refuse sex			0.008	
No	1,916 (14.0)	1359 (71.9)		1.00
Yes	12,577 (86.0)	9445 (74.9)		1.17 (1.04–1.31)
Beating justified if wife refuse sex with husband			0.305	
Yes	379 (2.8)	277 (71.8)		1.00
No	14,114 (97.2)	10,527 (74.6)		1.15 (0.87–1.51)
Respondent can ask to husband to use condom			<0.001	
No	2,671 (19.6)	1786 (67.9)		1.00
Yes	11,822 (80.4)	9018 (76.1)		1.50 (1.35–1.67)
Wife justified asking husband to use condom if he has a sexually transmitted infection				
No	373 (2.9)	263 (72.2)	0.304	1.00
Yes	14,120 (97.1)	10,541 (74.6)		1.13 (0.89–1.44)

BDHS, bangladesh demographic health survey; UOR, unadjusted odds ratio; CI, confidence interval.

^a^ In estimating percentages, the complex survey design and sampling weights were taken into account.

Results from Table 4 indicate that women’s sexual empowerment predicts contraceptive use among women, as both the bivariate and multivariate models show significant positive associations. A unit increase in the women’s sexual empowerment scale increases the unadjusted odds of contraceptive use by about 20% (UOR: 1.20, 95% CI: 1.14–1.28). However, the association remained the same after controlling for other sociodemographic variables. A unit increase in the sexual empowerment scale increases the odds of contraceptive use by 13% (adjusted OR: 1.13; 95% CI: 1.06–1.20). It is also observed that the adjusted odds of contraceptive use were 1.24 times (AOR: 1.24; 95% CI: 1.11–1.39) higher among women with secondary or a higher level of education compared to women with no or primary education and significantly lower for Muslim women compared to others (AOR: 0.66; 95% CI: 0.57–0.76). Additionally, compared to women who have no exposure, women who had exposure to radio less than once a week (AOR: 1.40; 95% CI: 1.04–1.89) and exposure to TV at least once a week (AOR: 1.32; 95% CI: 1.02–1.29) had higher odds of using contraception. Women living in rural environments were 13% less likely to use contraceptives than women living in urban environments. Additionally, there is also a strong positive association between the increasing number of living children and contraceptive use.

**TABLE 4:** Association between sexual empowerment and current contraceptive use, Bangladesh demographic and health survey, Bangladesh 2017–2018.

Characteristics	UOR (95% CI)	AOR (95% CI)
Sexual empowerment	1.20*** (1.14–1.28)	1.13*** (1.06–1.20)
Age (years)		
15–24	1.00	1.00
25–34	1.62*** (1.45–1.81)	0.59*** (0.50–0.69)
35–49	0.78*** (0.70–0.86)	0.24*** (0.20–0.29)
Educational attainment		
No or primary	1.00	1.00
Secondary or higher	1.24*** (1.15–1.35)	1.24*** (1.11–1.39)
Husband’s educational attainment		
No or primary	1.00	1.00
Secondary or higher	1.01 (0.93–1.10)	0.95 (0.85–1.06)
Religion		
Non-muslim	1.00	1.00
Muslim	0.75*** (0.66–0.85)	0.66*** (0.57–0.76)
No. of living children		
0	1.00	1.00
1	4.27*** (3.62–5.03)	5.04*** (4.26–5.95)
2	7.03*** (5.95–8.31)	14.19*** (11.58–17.40)
≥3	5.05*** (4.31–5.92)	17.41*** (14.07–21.55)
Age difference of the spouses, years		
<5	1.00	1.00
5–10	1.00 (0.90–1.12)	0.95 (0.85–1.07)
≥11	0.70*** (0.62–0.78)	0.69*** (0.61–0.78)
Household wealth		
Bottom quintile	1.00	1.00
Second quintile	0.89 (0.78–1.01)	0.90 (0.78–1.04)
Third quintile	0.84* (0.74–0.96)	0.87 (0.74–1.01)
Fourth quintile	0.95 (0.82–1.09)	0.98 (0.81–1.17)
Top quintile	0.89 (0.78–1.02)	0.92 (0.75–1.12)
Exposure to newspapers/magazine		
Not at all	1.00	1.00
Less than once a week	1.11 (0.92–1.33)	1.12 (0.92–1.38)
At least once a week	1.07 (0.86–1.31)	1.24 (0.98–1.59)
Exposure to radio		
Not at all	1.00	1.00
Less than once a week	1.23 (0.95–1.60)	1.40* (1.04–1.89)
At least once a week	1.02 (0.73–1.40)	1.31 (0.95–1.83)
Exposure to TV		
Not at all	1.00	1.00
Less than once a week	1.18 (0.99–1.39)	1.20* (1.00–1.44)
At least once a week	1.14** (1.03–1.25)	1.32* (1.02–1.29)
Current working status		
Not working	1.00	1.00
Working	1.20*** (1.10–1.31)	1.19*** (1.08–1.31)
Living environment		
Urban	1.00	1.00
Rural	0.91* (0.83–0.99)	0.87* (0.78–0.97)
Region		
Barisal	1.00	1.00
Chattogram	1.00 (0.83–1.21)	0.88 (0.72–1.07)
Dhaka	1.01 (0.85–1.21)	0.96 (0.79–1.18)
Khulna	0.92 (0.77–1.09)	0.91 (0.75–1.09)
Mymensingh	0.93 (0.77–1.1.4)	0.92 (0.75–1.12)
Rajshahi	0.96 (0.80–1.15)	0.95 (0.78–1.16)
Rangpur	1.15 (0.97–1.38)	1.04 (0.86–1.26)
Sylhet	0.75** (0.61–0.92)	0.71** (0.58–0.88)

BDHS, bangladesh demographic health survey; UOR, unadjusted odds ratio; AOR, adjusted odds ratio; CI, confidence interval.

*Significant at *p ≤ 0.05; **p ≤ 0.01; ***p ≤ 0.001.

## Discussion

This study assesses the relationship between women’s sexual empowerment and contraceptive behavior among married Bangladeshi women and finds that sexual empowerment is associated with contraceptive use with higher odds after controlling for other socio-demographic variables. The relationship between women empowerment and contraceptive use is well documented around the world [2, 6, 15–19], however, these studies focused on different dimensions of women’s empowerment except the sexual dimension. The use of sexual empowerment and its relationship to the use of contraceptives in Bangladesh make this study distinctive. Although we found only one study from Ghana in which women’s sexual empowerment was used to assess the use of contraceptives [20], to the best of our knowledge, this current study is the first in Bangladesh. Although more research is required to determine the cause-effect relationship between sexual empowerment and contraceptive use, verification of sexual empowerment measurement; the findings of the study imply that in assessing women’s contraception use, women’s sexual empowerment might play an important role.

Bangladeshi society is a typical example of patriarchy, which describes the distribution of power and resources within families in such a way that men maintain power and control over resources. Social roles, family responsibilities, and power dynamics within the family are significantly influenced by gender. In Bangladesh, as in other South Asian countries, men dominate the family and make decisions in the family as well as in personal relationships, leaving women powerless and reliant on them. A cautious analysis of social norms and behaviors related to gender, power relationships, and sexuality reveals that they are entrenched in the dominant social constructs of masculinity and/or the related norms on the need for the social control of women’s sexuality [27]. According to earlier research, women’s ability to express sexual agency and power in their relationships with their partners may be constrained by social norms about acceptable sexual behavior [28]. Women generally do not have the right to decide when to become pregnant or how many children to have. Additionally, they lack the authority to decide whether to end a pregnancy or use a contraceptive to control pregnancy. Our findings that more sexually empowered women are more likely to use contraceptives could be explained by the traditional characteristics of Bangladeshi women, who have relatively little control over their reproductive health outcomes and are less likely to talk about sexual matters with their husbands due to shyness, who lack sexual knowledge, and have little influence over household decisions [29]. Another possible reason might be the high prevalence of intimate partner violence (IPV) against women in Bangladesh; the lifetime prevalence of IPV is 72.6% (and a 12 months prevalence of 54%) [30]. If IPV is frequent in a community, women might not be able to negotiate safer sex with husbands or ask to use contraceptives for fear of violent reactions [31]. Furthermore, the concept of women’s empowerment encompasses three fundamental components of agency: choice, voice, and power [12], which enable them to bring about normative changes in gender dynamics and alter women’s roles within their families and communities [32]. These modifications entail corresponding alterations in the way men interact with women, including choices about the utilization of contraceptives. Empowered women have the potential to enhance their household status, promote their overall wellbeing, facilitate access to essential health-related services, and enable them to exercise agency in making decisions regarding contraception [33].

This study identified several other factors as having a significant association with contraceptive use. Current contraception use significantly decreases with increased age, Muslim religiosity, a larger age difference between spouses, and rural residency. Earlier findings suggest that the likelihood of using a contraceptive decreases with the increment of age [34, 35] which corroborates the results of the present study. Many older women may not be sexually active or have reduced coital frequency, which may account for their decreased use of contraception [36]. Consistent with prior research [18, 37], we found that there is a variation in the likelihood of using contraception with religious affiliation, with Muslims being less likely to use contraception than non-Muslims. Asian Muslims have higher levels of pro-natalist sentiments compared to their non-Muslim counterparts [37]. Despite the fact that Islam does not explicitly forbid the use of contraception, some people choose to abstain from using it for religious reasons [38, 39]. The current data revealed that a higher spouse age difference (>10 years) significantly affects current contraceptive use with lower odds, which is in line with other studies [40–42]. In spousal relationship, the age gap holds some meaning for partners, and the gaps more than 10 years tend to be regarded as non-normative, which might lead to marital dissatisfaction [41]. In an Indian study [42], it was noted that the age gaps of the spouses have an influence on marital stability, satisfaction, family size preference, and contraceptive use. The underlying reason may be that women with older husbands may have a lower social position, less control over resources and fewer life experiences. As a result, they may have less autonomy in the socioeconomic sphere, including in areas like education and economic activity, as well as in areas like freedom of speech and travel, control over childbearing, and the use of contraceptives [40, 43].

This study also reported that the odds of contraceptive use increase with the increased educational level of the women, consistent with prior research [18, 34, 44], suggesting that educating girls at least through the secondary level may be a useful strategy for boosting both the prevalence of contraceptive use and gender equity [45, 46]. Women who are educated are more likely than others to want smaller families and hence have a stronger motive to use contraception. Education also helps women’s reproductive control by elevating women’s status within the family [18]. This could be because educated women are more acquainted with formal institutions and health professionals, as well as better knowledgeable about available contraceptive methods and sources. Furthermore, once they have decided to control their fertility, educated women are more likely to use contraceptives effectively, with a reduced risk of cessation and failure [18].

Women’s working status, which has also been used as a surrogate for empowerment, was found to be significantly associated with contraceptive use in the present study, which suggests that the working status of women is an important contributor to using contraception. Consistent with previous research [44, 47, 48], this study found that radio and television exposure were associated with contraception use among the three different mass media included in this study. Exposure to radio and/or TV is a vital source of information that influences the current and/or future use of contraceptives by raising awareness and knowledge [49, 50]. Mass media, especially TV exposure, have an important impact on women’s empowerment, including the ability to make decisions regarding contraception, and women having media access can communicate more about safer sexual practices and the use of contraceptives [51]. It is indicated in the literature that soap operas are likely to have the greatest impact on altering values and behavior [48]. However, it is important to note that while there were significant differences in contraception use among women with varying levels of TV exposure (none, less than once a week, or at least once a week), the reported percentages did not exhibit substantial variation, which could be due to the utilization of a large sample size. Additionally, it is plausible that the higher prevalence of contraceptive utilization among women with no television exposure can also be attributed to their potential access to contraceptive information through lower levels of government healthcare establishments, such as home visits conducted by family welfare assistants (FWAs) or visits to community and satellite clinics [52].

The number of living children was found to be one of the strongest predictors of using contraception. With an increasing number of children, the odds of using contraception increase in dose-response fashion. This finding is consistent with previous studies across the globe [53, 54], including Bangladesh [18, 34]. In nulliparous women, the desired number of children is unfulfilled, the intention to conceive is strong, and they are less likely to use contraception. Women opt for contraception once they have achieved their intended family size. With the increase in the number of children, it is anticipated that their desired number of children will be achieved; consequently, they are more likely to use contraception. Consistent with prior research [18, 54], we observed that rural women are less likely to use contraception. Rural women may have less confidence in their decision-making abilities, a lower degree of autonomy, limited access to contraceptive methods, and lower living standards than their urban counterparts. In addition, rural women are susceptible to gender inequality, and males dominate female reproductive decisions [55]. In line with earlier studies in Bangladesh [56, 57], we also found regional variations in contraceptive use. Women living in the Sylhet division were found to be less likely to use contraception than women living in the Barisal division. Sylhet Division, located in the northeastern part of Bangladesh, is characterized by poor transportation infrastructure owing to its hilly terrain, geographical remoteness, prolonged ethnic conflicts, and social conservatism [58], all of which could be potential barriers to accessing contraceptive methods.

This study has some important limitations. Limited items that were included in the DHS were used for developing the sexual empowerment scale; therefore, this study was unable to include other aspects of sexual empowerment, such as the ability to express one’s sexuality and sexual desires [59]. The use of DHS data also limits our ability to conduct direct comparisons of women’s sexual empowerment to other related empowerment scales. However, the findings of this study suggest that sexual empowerment is distinct from general proxies of empowerment, which provide support to the theory of the multidimensional nature of empowerment [20]. In addition, there is some risk of social desirability bias as a result of questions being asked about sensitive topics like domestic violence and sexual activity. This study is based on the cross-sectional nature of DHS data. Therefore, the temporal relationship between women’s sexual empowerment and contraceptive use cannot be determined as the data for both variables were collected at the time of the survey. Finally, this quantitative study just presents the statistical relationship between women’s sexual empowerment and contraceptive use. Since women’s empowerment is a complex concept, in-depth qualitative studies along with quantitative research with more retrospective data are desirable to gain a comprehensive understanding of this multifaceted relationship.

Despite these limitations, the findings of this study suggest that even after adjusting for education, employment, mass media access, and other demographic variables associated with contraceptive use among currently married Bangladeshi women, the odds of contraceptive use significantly increase with the increment of the sexual empowerment score. Our findings suggest that future family planning programs might benefit from evaluating women’s perceptions of their sexual rights and options rigorously. Women with higher sexual empowerment might be able to overcome conservatism and initiate or participate in conversation with their husbands about sexual and reproductive health and contraceptive choices. Culturally sensitive programs for promoting women’s sexual empowerment could have strong implications for contraceptive use and achieving fertility intentions. It is crucial for family planning policymakers to address and assess women’s perceptions of their right to sexual and reproductive health and equity in order to advance towards obtaining universal access to reproductive health services.

## Data Availability

Data for this study were gathered from Demographic and Health surveys (DHS), which is freely available online at (https://dhsprogram.com).
