# Supplementary material for: Women’s Sexual Empowerment and Its Relationship to Contraceptive Use in Bangladesh: Findings From a Recent National Survey
Source: Int J Public Health. 2023 Oct 19;68:1606143. doi: 10.3389/ijph.2023.1606143 (PMC10620289; doi:10.3389/ijph.2023.1606143)
Supplement: Supplementary file 1 [file Table1.DOCX]

**Supplementary Table 1** Results of Tukey Honestly Significant Difference (HSD) test for multiple comparisons for mean differences of the sexual empowerment score across different study variables

| **Characteristics** | **Group vs Group** | **Group means** | | **Mean difference** | **HSD-test** |
| --- | --- | --- | --- | --- | --- |
| **Age (years)** | 15–24 vs. 25–34 | 3.7055 | 3.6931 | 0.0124 | 1.2497 |
|  | 15–24 vs. 35–49 | 3.7055 | 3.5355 | 0.1700 | 17.1535* |
|  | 25–34 vs. 35–49 | 3.6931 | 3.5355 | 0.1576 | 15.9038* |
| **No. of living children** | 0 vs. 1 | 3.6595 | 3.7296 | 0.0702 | 5.0794* |
|  | 0 vs. 2 | 3.6595 | 3.6789 | 0.0194 | 1.4049 |
|  | 0 vs. ≥3 | 3.6595 | 3.5343 | 0.1252 | 9.0642* |
|  | 1 vs. 2 | 3.7296 | 3.6789 | 0.0508 | 3.6746* |
|  | 1 vs. ≥3 | 3.7296 | 3.5343 | 0.1954 | 14.1436* |
|  | 2 vs. ≥3 | 3.6789 | 3.5343 | 0.1446 | 10.4691* |
| **Age difference of the spouses** | <5 vs. 5–10 | 3.6643 | 3.6568 | 0.0075 | 0.7371 |
|  | <5 vs. ≥11 | 3.6643 | 3.5642 | 0.1000 | 9.8472* |
|  | 5–10 vs. ≥11 | 3.6568 | 3.5642 | 0.0925 | 9.1101* |
| **Wealth Index** | Bottom quintile vs. 2^nd^ quintile | 3.5142 | 3.5776 | 0.0634 | 5.0904* |
|  | Bottom quintile vs. 3^rd^ quintile | 3.5142 | 3.6124 | 0.0982 | 7.8822* |
|  | Bottom quintile vs. 4^th^ quintile | 3.5142 | 3.6590 | 0.1448 | 11.6257* |
|  | Bottom quintile vs. 5^th^ quintile | 3.5142 | 3.7735 | 0.2593 | 20.8158* |
|  | 2^nd^ quintile vs. 3^rd^ quintile | 3.5776 | 3.6124 | 0.0348 | 2.7918 |
|  | 2^nd^ quintile vs. 4^th^ quintile | 3.5776 | 3.6590 | 0.0814 | 6.5353* |
|  | 2^nd^ quintile vs. 5^th^ quintile | 3.5776 | 3.7735 | 0.1959 | 15.7255* |
|  | 3^rd^ quintile vs. 4^th^ quintile | 3.6124 | 3.6590 | 0.0466 | 3.7435 |
|  | 3^rd^ quintile vs.5^th^ quintile | 3.6124 | 3.7735 | 0.1611 | 12.9336* |
|  | 4^th^ quintile vs. 5^th^ quintile | 3.6590 | 3.7735 | 0.1145 | 9.1902* |
| **Exposure to newspapers/ magazines** | Not at all vs. less than once a week | 3.6096 | 3.7872 | 0.1776 | 8.6591* |
|  | Not at all vs. at least once a week | 3.6096 | 3.8632 | 0.2536 | 12.3648* |
|  | Less than once a week vs. at least once a week | 3.7872 | 3.8632 | 0.0760 | 3.7056* |
| **Exposure to radio** | Not at all vs. less than once a week | 3.6236 | 3.7883 | 0.1647 | 5.4010* |
|  | Not at all vs. at least once a week | 3.6236 | 3.8070 | 0.1835 | 6.0159* |
|  | Less than once a week vs. at least once a week | 3.7883 | 3.8070 | 0.0188 | 0.6149 |
| **Exposure to TV** | Not at all vs. less than once a week | 3.5457 | 3.5680 | 0.0224 | 1.7311 |
|  | Not at all vs. at least once a week | 3.5457 | 3.6991 | 0.1534 | 11.8792* |
|  | Less than once a week vs. at least once a week | 3.5680 | 3.6991 | 0.1311 | 10.1481* |
| **Region** | Barisal vs. Chattogram | 3.6537 | 3.5837 | 0.0700 | 4.3827* |
|  | Barisal vs. Dhaka | 3.6537 | 3.6909 | 0.0372 | 2.3293 |
|  | Barisal vs. Khulna | 3.6537 | 3.6517 | 0.0020 | 0.1264 |
|  | Barisal vs. Mymensingh | 3.6537 | 3.6771 | 0.0234 | 1.4668 |
|  | Barisal vs. Rajshahi | 3.6537 | 3.6076 | 0.0461 | 2.8858 |
|  | Barisal vs. Rangpur | 3.6537 | 3.6134 | 0.0403 | 2.5239 |
|  | Barisal vs. Sylhet | 3.6537 | 3.5606 | 0.0931 | 5.8304* |
|  | Chattogram vs. Dhaka | 2.5837 | 3.6909 | 0.1072 | 6.7120* |
|  | Chattogram vs. Khulna | 2.5837 | 3.6517 | 0.0680 | 4.2563 |
|  | Chattogram vs. Mymensingh | 2.5837 | 3.6771 | 0.0934 | 5.8494* |
|  | Chattogram vs. Rajshahi | 2.5837 | 3.6076 | 0.0239 | 1.4968 |
|  | Chattogram vs. Rangpur | 2.5837 | 3.6134 | 0.0297 | 1.8588 |
|  | Chattogram vs. Sylhet | 2.5837 | 3.5606 | 0.0231 | 1.4477 |
|  | Dhaka vs. Khulna | 3.6909 | 3.6517 | 0.0392 | 2.4557 |
|  | Dhaka vs. Mymensingh | 3.6909 | 3.6771 | 0.0138 | 0.8626 |
|  | Dhaka vs. Rajshahi | 3.6909 | 3.6076 | 0.0833 | 5.2152* |
|  | Dhaka vs. Rangpur | 3.6909 | 3.6134 | 0.0775 | 4.8532* |
|  | Dhaka vs. Sylhet | 3.6909 | 3.5606 | 0.1303 | 8.1597* |
|  | Khulna vs. Mymensingh | 3.6517 | 3.6771 | 0.0254 | 1.5931 |
|  | Khulna vs. Rajshahi | 3.6517 | 3.6076 | 0.0441 | 2.7595 |
|  | Khulna vs. Rangpur | 3.6517 | 3.6134 | 0.0383 | 2.3975 |
|  | Khulna vs. Sylhet | 3.6517 | 3.5606 | 0.0911 | 5.7040* |
|  | Mymensingh vs. Rajshahi | 3.6771 | 3.6076 | 0.0695 | 4.3526* |
|  | Mymensingh vs. Rangpur | 3.6771 | 3.6134 | 0.0637 | 3.9906 |
|  | Mymensingh vs. Sylhet | 3.6771 | 3.5606 | 0.1166 | 7.2971* |
|  | Rajshahi vs. Rangpur | 3.6076 | 3.6134 | 0.0058 | 0.3620 |
|  | Rajshahi vs. Sylhet | 3.6076 | 3.5606 | 0.0470 | 2.9445 |
|  | Rangpur vs. Sylhet | 3.6134 | 3.5606 | 0.0528 | 3.3065 |

**p*<0.05
